# Mental health and psychosocial well-being during the COVID-19 pandemic: the invisible elephant in the room

**DOI:** 10.1186/s13033-020-00371-w

**Published:** 2020-05-28

**Authors:** Akaninyene Otu, Carlo Handy Charles, Sanni Yaya

**Affiliations:** 1 Department of Infection and Travel Medicine, Leeds Teaching Hospitals NHS Trust, Leeds, UK; 2 Department of Internal Medicine, College of Medical Sciences, University of Calabar, Calabar, Cross River State Nigeria; 3 Department of Sociology, McMaster University, Hamilton, ON Canada; 4 Geography at the Laboratoire Caribéen de Sciences Sociales du Centre National de la Recherche Scientifique, Université des Antilles, Schoelcher, Martinique, France; 5 School of International Development and Global Studies, University of Ottawa, Ottawa, ON Canada; 6 The George Institute for Global Health, The University of Oxford, Oxford, UK

**Keywords:** Mental Health, Psychosocial well-being, Covid-19, Pandemic, Outbreak, Health inequalities

## Abstract

The novel SARS-CoV-2 coronavirus pandemic has emerged as a truly formidable threat to humankind’s existence. In the wake of the massively volatile global situation created by COVID-19, it is vital to recognize that the trauma it causes can affect people in different ways, at the individual and collective levels, resulting in mental health challenges for many. Although mental health problems account for about one-third of the world’s disability among adults, these issues tend to be under-addressed and overlooked in society and are closely associated with deadly disease outbreaks. In large scale outbreaks, the mental health problems experienced are not limited to infected persons but also extend to involve frontline health workers and community members alike. While it is crucial to limit the spread of infections during an outbreak, previous experience suggests that mental and behavioural health interventions should be fully included in public health response strategies.

## Background

As the novel SARS-CoV-2 coronavirus pandemic sweeps rapidly across the globe, it is undoubtedly having immense psychological impacts on communities. Some of these impacts can be linked to elevated levels of stress or anxiety [1]. There are legitimate concerns that an epidemic of mental illness could actually occur in the midst of the current COVID-19 pandemic and affect all generations and all majority and minority groups–albeit differently [2]. Measures such as quarantine and physical distancing, while vital to stemming transmission of COVID-19, may contribute to a rise in depression, self-harm, harmful alcohol and drug use with further negative psychological consequences. Infectious disease outbreaks have also been associated with stigma and xenophobia [3]. Socioeconomic disparities resulting from job losses and other systemic barriers can exacerbate mental health issues among the general population amid COVID-19. In this regard, primary considerations should be given to how we guarantee service access and continuity for persons with existing mental health conditions whilst protecting the mental wellbeing of frontline workers, persons with COVID-19, and the general population [1].

In a recent survey carried out among 775 adults in the United States, an alarming 55% of them admitted that COVID-19 has had deleterious effects on their mental wellbeing and 71% were worried about the potential negative impact of isolation on the mental health of Americans [1]. Substance abuse and mental health hotline services across the United States have registered unprecedented increases in call volumes since February 2020 [4]. Although mental health problems account for about one-third of the world’s disability among adults, these issues tend to be under-addressed and overlooked in society [5]. A peculiarity of mental health management is that solutions need to be consistently applied to be successful and there is little room for “quick fixes” with these issues. Therefore, the institution of accessible and sustainable community-wide support systems would be the preferred approach to addressing mental health challenges in this pandemic. However, the global focus is currently on implementing measures of physical distancing whilst caring for unwell persons with COVID-19 in hospitals and ensuring that ventilators are available to support the sick in intensive care settings. Issues such as mental health of the general population, frontline health workers and persons with COVID-19 are being overlooked.

Although the actual scale of the mental health burden of COVID-19 is only beginning to emerge, the effects are likely to remain long after the pandemic has been dealt with. In many ways, the threat to people’s mental health appears to be invisible and ambiguous, one which requires urgent attention nonetheless [4]. The description of the mental health problems associated with COVID-19 as a “slow-motion disaster” is very apt as the psychological fallouts are likely to be widespread and persistent [4]. With lockdowns and physical distancing strategies being imposed in most nations, people have turned to different media sources to get news updates on the pandemic. Accessing these information sources for prolonged periods of time is very likely to increase anxiety and potential for hysteria thus impacting one’s mental wellbeing.

### When hard-pressed frontline health workers are putting their patients’ needs before their own, it’s time to care for their mental health

Frontline health workers are faced with the daunting task of caring for large numbers of very sick persons in the midst of uncertainty and upheaval. With every passing day, the personal protective equipment (PPE), laboratory testing and other resources needed to protect healthcare workers from COVID-19 infection are becoming increasingly scarce. The anxiety of working in such pressured environments and lack of rest can indirectly increase the probability of acquiring COVID-19 from working in health facilities. The fear of contracting a contagion can negatively impact the willingness of health workers to provide service in outbreak situations. In a survey carried out among 308 public health workers in Maryland USA, nearly half of them were not likely to report to duty during a pandemic due to the perceived risks of infection [6]. Such perceptions are likely to negatively impact a health system’s readiness to deal with pandemics such as COVID-19. Previous disease outbreaks such as severe acute respiratory syndrome (SARS) in 2003 and Ebola disease in 2014–2016 were associated with a spectrum of mental health problems among both the general population and health workers such as depression, anxiety, fear, post-traumatic stress disorder and frustration [7–9].

Health workers are characteristically resilient and constitute the first line of defense in the face of disease outbreaks. They carry out their life-saving duties at great personal risk and this sometimes entails isolating themselves from family members to limit the risk of spreading a contagion. Underneath the façade of calm these health workers typically project lies a vulnerability characterized by anxiety, fear of contracting an infectious disease and anticipation of impending doom [10]. These tensions are further magnified by fear of being quarantined or having to deal with inadequacies on the system such as a lack of PPE and ventilators. Some health workers have described feeling coerced and betrayed by their employers and the government in outbreak situations [10]. There are also the added anxieties involved with the assumption of new or unfamiliar clinical roles in outbreak situations. When interviewed, health workers involved in pandemics described feeling afraid, lonely, ostracized and unhappy about restrictions on behaviours that enhanced coping, such as attending social events and physical contact [11].

The term “vicarious trauma” was first described in 1996 [12] and was initially applied to situations where psychotherapists became affected by long-term contact with patients with mental diseases and manifested mental symptoms similar to psychological trauma [13]. The term has since been extended to include sympathy for survivors of a trauma resulting in physical symptoms such as loss of appetite, irritability, fatigue and sleep disorders. Given the devastating nature of the current COVID-19 pandemic, it is very likely that vicarious trauma is occurring in healthcare settings dealing with the COVID-19 pandemic [14]. A study conducted among over 1200 health care workers providing care to COVID-19 patients in China reported higher levels of severe mental health symptoms in the frontline workers when compared to those in secondary roles [15]. The COVID-19 response has been characterized by an unprecedented global market failure in the supply of PPE to health workers, which has been linked to the unacceptably high infection rates among health workers [16]. There have also been reports of overwork by nurses caring for COVID-19 patients without adequate provision being made for their recuperation and mental wellbeing [16]. Patients infected with similar infections do not appear to be spared mental problems as depression, suicidality, anxiety, panic attacks and delirium have been reported among such cohorts [7, 17].

### The COVID-19 pandemic: an opportunity to strengthening mental health systems and policies

In response to the threat of death from COVID-19, the world has experienced massive shifts and re-organization at a collective level with potential for even greater social transformation [18]. There are arguments that the COVID-19 pandemic might be leveraged to merge public health with mental health, foster togetherness and reduce prejudice and stigma [18]. In a bid to curb community transmission of COVID-19, many national governments have resorted to activating emergency measures, such as imposition of lockdowns, closure of schools, self-isolation, limiting the people in public places and at ceremonies, such as weddings and burials to ensure physical distancing. The family and friends of persons with COVID-19 who get admitted in hospitals are not being allowed to visit their sick loved ones out of fear of transmitting the virus. These strategies, which limit normal human interaction–combined with fear of the consequences of infection and social media misinformation–increase the levels of chaos, stress and tensions within communities. The additional stressors such as loss of loved ones due to COVID-19, economic anxiety from job losses and financial instability all constitute clear and present dangers. Isolation and physical seclusion are reportedly associated with increase in issues such as child abuse, intimate-partner violence and suicide [7, 19].

Specific legislations are needed, at national and local levels, to mitigate the mental health fallouts of COVID-19 and strengthen existing community mental health programmes. There is a need for both horizontal and vertical integration of these policies. The horizontal aspect should involve the establishment of comprehensive mental health services while vertical integration should promote the alignment of these services with relevant social, economic, and political policy domains [20]. Governance frameworks should be put in place to address mental health literacy and achieve stigma reduction [21]. Prioritization of mental health and deliberate appropriation of financial resources to tackle mental health issues by policy makers are crucial measures. Access to publicly funded mental health therapy should be widely promoted. In this vein, the Australian government recently released a $1.1 billion package to boost mental health services to the Australian people battling the devastating impacts of COVID-19 [22]. This represents a positive step in the right direction.

The human resources that provide mental health services should also be augmented and this could be achieved by task-sharing [23]. Specific short and medium-term training programmes targeting general health workers could be rolled out. This would bolster capacity to provide sustainable and integrated mental health services at primary health care level during and after the COVID-19 pandemic. There may also be a need to build in a robust health management information system (HMIS) integrated with mental health indicators to facilitate the monitoring of mental healthcare within the communities [23]. Those with serious mental health illnesses are particularly vulnerable at this time and safeguards should be put in place to protect them [21].

### Addressing Socio-economic disparities in mental health: The need to protect underserved and vulnerable populations

Existing evidence has clearly established a link between socio-economic characteristics (ethnicity, gender, social position, educational and wealth status and standard of living, etc.) and rates mental health service utilization [24]. There is a pressing need to address unequal access to healthcare as it is well known that social stratification produces all types of inequality around the world [25]. For instance, in Western countries, the most vulnerable people tend to be those without permanent legal status, such as asylum seekers, temporary foreign nationals [26], and other poor, marginalized citizens [27] with limited or no access to healthcare [28, 29]. During pandemics, these vulnerable populations and the frontline health workers with whom they interact face a considerable challenge to help these foreign nationals who may not master the local language or may not be familiar with how to access available resources. Social research needs to be conducted to shed light on how socioeconomic disparities affect mental health amid the COVID-19 pandemic in comparison to past pandemics [27, 28].

Since the COVID-19 outbreak in the United States, emerging data have suggested that black Americans are disproportionately impacted by the virus [30]. This is mirrored by high mortality rates in states such as Louisiana, New Orleans, Chicago Detroit, where black Americans constitute a majority of the population [30]. Similar imbalances are also being seen in Midwestern cities such as Detroit and Chicago. Even in states such Illinois and Milwaukee where black people represent 14.6% and 26 percent of the population respectively, black people made up almost half and 42% percent of fatalities respectively [31]. Some of the reasons attributed to this disparity include the high prevalence of chronic diseases, which predispose to death with COVID-19 disease among back Americans, health inequity and racial disparity [31].

Research intersecting socio-economic inequalities and health can be used to understand why black Americans are disproportionately being affected by the COVID-19 pandemic as opposed to other ethno-racial groups. Indeed, overwhelming evidence suggests that socioeconomic inequalities–meaning inequalities that relate to differences in income, social class, occupational background, educational achievement, and neighbourhood deprivation can significantly impact people’s health outcomes [32]. In the United States, socioeconomic inequalities leave many poor black Americans with no choice but to use public transportation and take up roles such as cleaning in hospitals, for instance, which puts them at greater risk of acquiring COVID-19 [31]. Rigorous quantitative and qualitative social research needs to be carried out in order to shine light on the multidimensional factors that may account for this increased trend of COVID-19-related mortality among black Americans.

### Expanding mental and behavioural health measures in a time of pandemic matters more than ever

The World Health Organization has recently put out guidance on mental health and psychosocial considerations during the COVID-19 pandemic, which specifically targets healthcare workers, the general population, those in isolation and people with co-morbidities [33]. For health workers, education on both appropriate use of PPE and key principles underpinning the management of suspected/confirmed COVID-19 patients is advocated. Health workers should be trained to identify early signs of undue stress/burnout at work and seek help immediately. Other practical measures include incorporating rotations from higher-to lower stress tasks and team “huddles” to ease tensions within the workplace. Regular clinical screening for anxiety, depression and other mental health issues might be useful for both health workers and patients infected with COVID-19. Multidisciplinary mental health and crisis teams will need to be constituted and made accessible by health workers, patients and community members. Provision of psychological counselling hotlines and online channels may help to boost access to such teams by the above groups.

As for the general population, the World Health Organization recommends that we treat people who are affected by COVID-19 with compassion and kindness; and that we separate a person from having an identity defined by COVID-10-in order to reduce stigma. The WHO advises that we curb our exposure to COVID-19-related news that can cause anxiety and psychological distress. Instead, the WHO suggests that we get informed by trusted persons while focusing on facts rather than misinformation and rumours. In addition to protecting ourselves, we should also be supportive to others by amplifying positive and hopeful stories and positive images of local people who have experienced COVID-19. Finally, the WHO advises the general population to honour carers and healthcare workers by acknowledging the role they place in saving lives and keeping our loved ones safe [33].

In this highly critical time, it is important that everybody does their part to slow the spread of COVID-19 by following governments’ public health measures of physical distancing as well as the above-mentioned WHO recommendations. In doing so, social media and other communication technologies can be our allies. Social media, for example, can be a useful tool for information dissemination, which can be leveraged to share strategies for dealing with psychological stress in the society. General measures such as leading active lifestyles, eating healthily and maintaining social links via a number of communication platforms should be encouraged. The establishment of community coalitions and networks to support the elderly and infirm who may be unable to cope with the isolation measures should be given priority.

## Conclusion

The COVID-19 pandemic is putting an enormous strain on the health care systems. It is not only a medical crisis. Given the apocalyptic speed with which COVID-19 is sweeping across the globe, the mental health care of patients, health professionals and communities is very likely to be under-addressed thus giving rise to major medium and long-term consequences. The establishment of mitigating strategies to preserve the mental wellbeing of all is a complicated, but crucial imperative. A proactive and multipronged approach comprising longer term strategies rather than short-term crisis responses is desirable. Inaction in the face of this under-recognized threat is likely to have grave consequences for humankind.

## Data Availability

Data sharing is not applicable to this article as no datasets were generated or analyzed during the current study.
